# Taiwan Society of Colon and Rectal Surgeons Consensus on mCRC Treatment

**DOI:** 10.3389/fonc.2021.764912

**Published:** 2021-11-15

**Authors:** Hong-Hwa Chen, Tao-Wei Ke, Ching-Wen Huang, Jeng-Kae Jiang, Chou-Chen Chen, Yao-Yu Hsieh, Hao-Wei Teng, Bo-Wen Lin, Yi-Hsin Liang, Yu-Li Su, Hung-Chih Hsu, Feng-Che Kuan, Yenn-Hwei Chou, Johnson Lin, Ben-Ren Lin, Yu-Yao Chang, Jaw-Yuan Wang

**Affiliations:** ^1^ Division of Colorectal Surgery, Department of Surgery, Chang Gung Memorial Hospital, Kaohsiung, Taiwan; ^2^ Department of Colorectal Surgery, China Medical University Hospital, Taichung, Taiwan; ^3^ Division of Colorectal Surgery, Department of Surgery, Kaohsiung Medical University Chung-Ho Memorial Hospital, Kaohsiung, Taiwan; ^4^ Department of Surgery, College of Medicine, Kaohsiung Medical University, Kaoshiung, Taiwan; ^5^ Division of Colon and Rectal Surgery, Department of Surgery, Veterans General Hospital, Taipei, Taiwan; ^6^ Department of Surgery, Veterans General Hospital, Taichung, Taiwan; ^7^ Division of Hematology and Oncology, Taipei Medical University-Shuang Ho Hospital, Ministry of Health and Welfare, New Taipei City, Taiwan; ^8^ Division of Medical Oncology, Department of Oncology, Veterans General Hospital, Taipei, Taiwan; ^9^ Department of Surgery, National Cheng Kung University Hospital, Tainan, Taiwan; ^10^ Department of Oncology, National Taiwan University Hospital, Taipei, Taiwan; ^11^ Division of Hematology Oncology, Department of Internal Medicine, Chang Gung Memorial Hospital, Kaohsiung, Taiwan; ^12^ Division of Hematology Oncology, Chang Gung Memorial Hospital, Linkou, Taiwan; ^13^ Department of Hematology and Oncology, Chang Gung Memorial Hospital, Chiayi, Taiwan; ^14^ Division of General Surgery, Department of Surgery, Shin-Kong Wu Ho Su Memorial Hospital, Taipei, Taiwan; ^15^ Division of Hematology and Oncology, MacKey Memorial Hospital, Taipei, Taiwan; ^16^ Department of Colorectal Surgery, Changhua Christian Hospital, Changhua, Taiwan; ^17^ Graduate Institute of Clinical Medicine, College of Medicine, Kaohsiung Medical University, Kaoshiung, Taiwan; ^18^ Pingtung Hospital, Ministry of Health and Welfare, Pingtung, Taiwan

**Keywords:** Taiwan, metastatic colorectal cancer, treatment consensus, molecular biomarker, disease management in real world practice

## Abstract

Therapeutic options for metastatic CRC (mCRC) have changed significantly in recent years, greatly increasing the complexity of therapeutic decision-making. Although oncology guidelines have helped improve the care process, guidelines may also limit the flexibility to individualize in-clinic decision-making. This consensus paper addresses specific gaps in the current international guidelines to assist Taiwanese colon and rectal experts make specific therapeutic choices. Over 3 years and three meetings with selected experts on “real-world” Taiwanese practice patterns for mCRC, consensus was achieved. The experts also discussed specific questions during in-depth one-on-one consultation. Outcomes of the discussion were then correlated with published evidence by an independent medical writer. The final consensus includes clinically implementable recommendations to provide guidance in treating Taiwanese mCRC patients. The consensus includes criteria for defining fit and unfit intensive treatment patients, treatment goals, treatment considerations of molecular profiles, treatment consideration, and optimal treatment choices between different patient archetypes, including optimal treatment options based on *RAS*, *BRAF*, and microsatellite instability (MSI) status. This consensus paper is the second in the Taiwan Society of Colon and Rectal Surgeons (TSCRS) Consensus series to address unmet gaps in guideline recommendations in lieu of Taiwanese mCRC management. Meticulous discussions with experts, the multidisciplinary nature of the working group, and the final drafting of the consensus by independent medical professionals have contributed to the strong scientific value of this consensus.

## Introduction

### Epidemiology of mCRC

Colorectal cancer (CRC) represents 10% of global cancer incidence and 13% of all-cause deaths (1). It is the second most common cancer in women and the third most common cancer in men. It occurs more commonly in developed countries, but the mortality rate is higher in developing countries (2).

In Taiwan, more than 16,400 new CRC cases occurred in 2017 with an incidence rate of 69.61 per 100,000, mortality rate of 24.66 per 100,000 (3), 5-year survival rate of 63.0% (2015), and mean survival time after CRC diagnosis of 71.27 ± 1.27 months (4). Because mCRC incidence is increasing rapidly in Taiwan, it is critical for experts to examine new methods to provide patients with the latest therapy, optimal survival, and acceptable quality of life.

### Differences Between NCCN, ESMO, and Pan-Asian ESMO Guidelines

Oncology guidelines have helped improve both cancer care and patient outcomes (5). The most appreciated and widely used comprehensive guidelines include the European Society for Medical Oncology (ESMO) Colorectal Cancer Guidelines, the National Comprehensive Cancer Network (NCCN) Guidelines in Colorectal Cancer, and the Pan- Asian adapted ESMO consensus guidelines for the Asian region. Several differences are noted when comparing these guidelines (**Table 1**). (1) ESMO and Pan-Asia ESMO guidelines further stratify treatment by different treatment goals, but NCCN guidelines do not. (2) Tumor-sidedness is not considered in ESMO guidelines. (3) Pan-Asia ESMO guidelines recommend doublet CT plus anti-EGFR in all *RAS* WT left-sided patients; NCCN guidelines recommend both anti-EGFR and anti-VEGF in these patients. (4) Pan-Asia ESMO guidelines consider doublet CT plus anti-EGFR in *RAS* WT right-sided patients when cytoreduction is the goal, but NCCN guidelines do not. (5) NCCN guidelines have updated encorafenib plus anti-EGFR in 2L *BRAF* mutant patients. (6) Pan-Asia ESMO guidelines and NCCN guidelines recommend immunotherapy for patients with dMMR/MSI- H mCRC at different levels of recommendation.

**Table 1:** Treatment recommendations for first-line management of mCRC in NCCN, ESMO, and Pan-Asian guidelines.

Guidelines	Treatment Goal	Left-sided *RAS* WT	Right-sided *RAS* WT	*RAS* MUT	*RAS* WT/*BRAF*MUT	dMMR/MSI-H
**ESMO** **2016 (** 6 **)**	**Cytoreduction**	Doublet CT plus anti-EGFR	Doublet CT plus anti-EGFR	Combination CT plus Bev	Triplet CT plus Bev	Not applicable
	**Disease Control**	Doublet CT plus biological agent	Doublet CT plus biological agent	Doublet plus Bev	Triplet CT ± Bev	Not applicable
**Pan-Asian** **ESMO** **2018/2020(** 7 **;** 8 **)**	**Cytoreduction**	Doublet CT plus anti-EGFR	• Triplet/doublet CT ± Bev • Doublet CT plus anti-EGFR if the goal is tumor size reduction	Combination CT plus Bev	Triplet CT plus Bev	Immunotherapy (when no other satisfactory treatment option exists depending on the clinical context)
	**Disease Control**	Doublet CT plus anti-EGFR	Doublet CT ± Bev	Doublet CT plus Bev	Triplet CT plus Bev	
**NCCN** **2021(1.2021).** **(** 9 **)**	**Patient fit for** **Intensive Therapy**	• Doublet CT plus anti-EGFR • Triplet/doublet CT ± Bev	Triplet/doublet CT ± Bev	Triplet/doublet CT ± Bev	• 1L: Triplet/ doublet CT ± Bev • 2L: Encorafenib plus anti-EGFR (*BRAF*V600E)	• Pembrolizumab • Nivolumab ± Ipilimu mab (Patients should be followed closely for 10 weeks to access for response)

### Need for a Taiwanese Expert Consensus

Differences are found between regions of Taiwan regarding issues ranging from patient selection to treatment approaches. Therefore, incorporating local practice must be considered when attempting to standardize and improve treatment for mCRC patients locally. Accordingly, Taiwanese surgical and oncology leaders with extensive experiences in treating mCRC have collaborated to address key areas in the current mCRC treatment landscape and have reached consensus on recommendations for different treatment strategies.

## Methodology

This consensus included synchronous and metachronous colon and rectal cancer patients with any T, any N, and M1. The consensus procedure performed was similar to the Delphi method. The following key characteristics of the Delphi method were applied to help establish this consensus including anonymous voting, structural information, and regular feedback. The participating experts were selected based on the following criteria: (1) demonstrated knowledge/expertise in CRC and (2) geographic representation of the North, Central, and South Taiwan. Consensus was achieved between colorectal experts over the course of three advisory board meetings in 2018, 2019, and 2020 on “real-world” Taiwanese practice patterns for mCRC. During meetings, evidence-based algorithms were generated schematically by incorporating updated scientific evidence and referring to existing NCCN, ESMO, and Pan-Asian ESMO guidelines. Discussion followed a list of questions that had been prepared and selected before the meeting during the one-on-one consultation, and the recommendations were based on the level of consensus achieved after participating experts voted. The voting process was anonymous.

Additional one-on-one consultations lasting 30–40 min each were performed to collect additional opinions and recommendations from individual experts. The experts responded to each recommendation with “agree” or “disagree.” To represent the consensus, each recommendation was required to have the agreement of at least two-thirds of the experts. Outcomes of these meetings were further contextualized by an independent medical writer through reviewing published literature. This consensus paper is an independent report of the expert panel and is not a policy statement of the Taiwan Society of Colon and Rectal Surgeons (TSCRS). This is the second treatment consensus from TSCRS; the preceding development of TSCRS Consensus for Cytoreduction Selection in Metastatic Colorectal Cancer in 2017 has been published on the *Annals of Surgical Oncology* (10).

**Figure 1:**
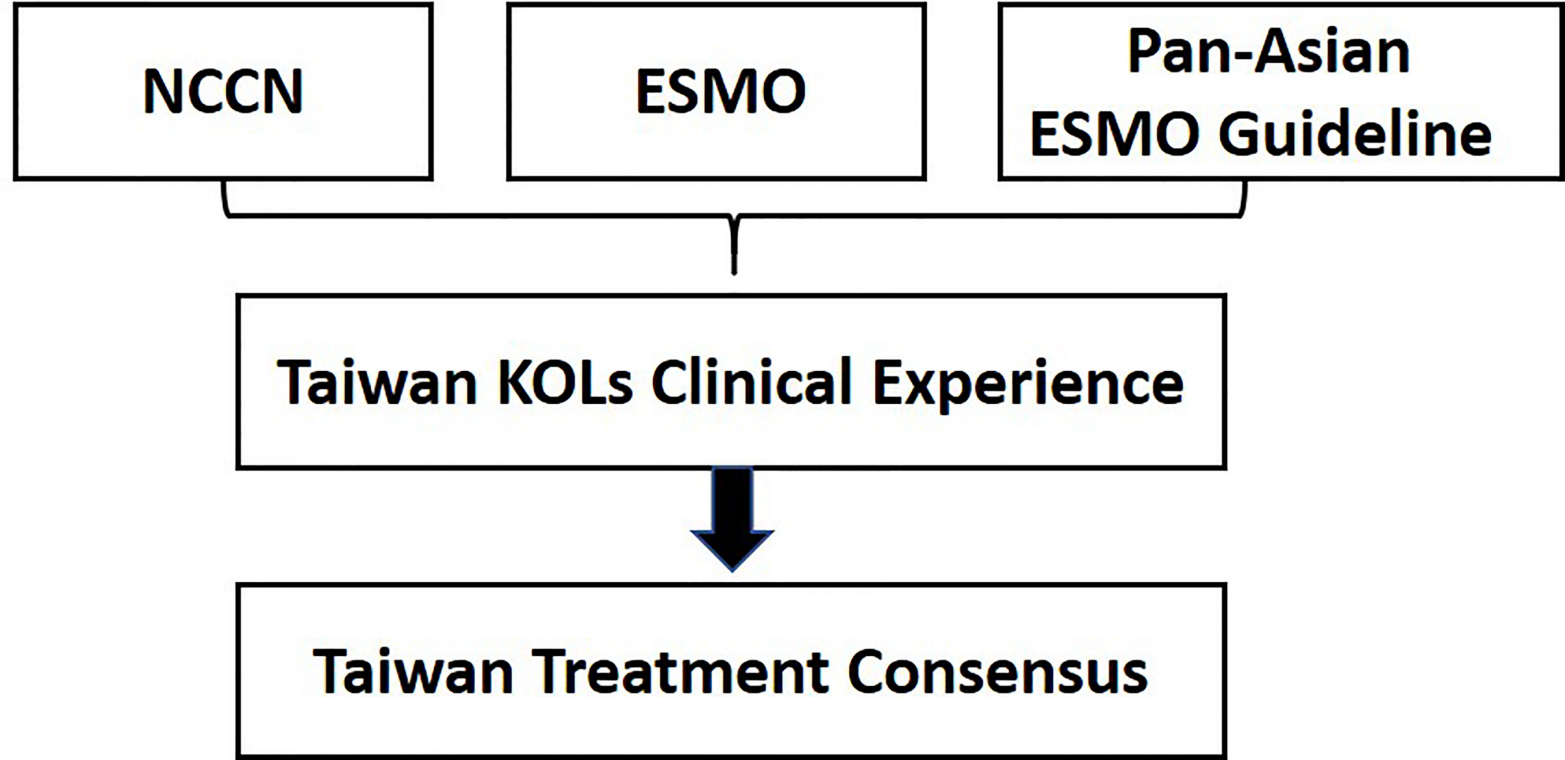
Approach of Consensus Formation.

The basic approach of consensus formation is shown in **Figure 1** and **Table 2**, respectively. The first meeting was held in August 2018, and six experts deliberated on four key recommendations. The second meeting was held in January 2019 with six experts discussing four more recommendations. The last meeting took place in July 2020 including 6 core members who presented in the first two meetings and additional 12 experts to conclude and revalidate the findings of the previous meetings from the standpoint of the latest guidelines and updated clinical evidence in the new area of microsatellite instability-high (MSI-H) or mismatch repair deficient (dMMR) and mutated *BRAF* genes. The expert panel structure and objectives of the meetings held over 4 years are presented in **Table 3**. The recommendations are based only on the locally approved agents and biologicals in Taiwan.

**Table 2:** Summary of recommendations.

Question	Recommendation
**Q1. What is the definition of “fit” and “unfit” patients?**	• Patients should be assessed as fit or unfit based on medical condition, not malignant disease. • Multiple factors, including age, performance status, organ function, comorbidities, and patient attitude should be considered. Level of agreement: 100% (17/17) Quality of evidence: VA
**Q2. What should be the initial treatment goal for mCRC?**	• For fit patients with resectable metastatic disease, the treatment goal is curative intent; for fit patients with unresectable metastatic disease, the treatment goal is either cytoreduction or disease control. Level of agreement: 100% (17/17) Quality of evidence: VA
**Q3. Which biomarker analysis should be considered before initial treatment of mCRC?**	• *RAS*, *BRAF*, and MMR/MSI testing should be done before first-line systemic treatment. • For MMR/MSI testing, both PCR and IHC can be adopted. Level of agreement: 100% (17/17) Quality of evidence: IA
**Q4. When cytoreduction is the goal, what is the initial treatment recommendation for patients with *RAS*/*BRAF*** **wt mCRC?**	• When cytoreduction is the goal, CT doublet plus anti-EGFR is recommended as 1L treatment for patients with *RAS*/*BRAF* wt mCRC, regardless of primary tumor location. Level of agreement: 82% (14/17) Quality of evidence: IIA
**Q5. When disease control is the goal, what is the initial treatment recommendation for patients with *RAS*/*BRAF* wt mCRC?**	• When disease control is the goal, CT doublet plus anti-EGFR is recommended as 1L treatment for patients with left-sided, *RAS*/*BRAF* wt mCRC. Quality of evidence: IA • When disease control is the goal, CT doublet plus anti-VEGF is recommended as 1L treatment for patients with right-sided, *RAS*/*BRAF* wt mCRC. Quality of evidence: IA Level of agreement: 94% (16/17)
**Q6. What is the initial treatment recommendation for patients with *RAS* mt mCRC?**	• When cytoreduction is the goal, CT doublet/triplet plus anti- VEGF is recommended as 1L treatment for patients with *RAS* mt mCRC. Quality of evidence: IIA • When disease control is the goal, CT doublet plus anti-VEGF is recommended as 1L treatment for patients with *RAS* mt mCRC. Quality of evidence: IB Level of agreement: 100% (17/17)
**Q7. What is the initial treatment recommendation for patients with *BRAF* mt** **mCRC?**	• Both CT triplet/doublet plus anti-VEGF could be considered for *BRAF* mutation patients based on patients’ tolerability. Level of agreement: 82% (14/17) Quality of evidence: IIB
**Q8. What is the second-line treatment recommendation for patients with *BRAF* mt** **mCRC?**	• BRAFi plus anti-EGFR ± MEKi is recommended as the second-line treatment for patients with *BRAF* mt mCRC. Level of agreement: 100% (17/17) Quality of evidence: IA
**Q9. What is the treatment recommendation for patients with dMMR/MSI-H mCRC?**	• In addition to CT plus target therapy, the immuno-checkpoint inhibitor is an alternative option for any line of treatment for patients with dMMR/MSI-H mCRC. Level of agreement: 100% (17/17) Quality of evidence: IC

**Table 3:** Structure and objectives of three expert panel meetings.

		Expert Panel Meetings	
Objective	Treatment	Treatment	Revisit previous
	recommendations for	recommendation for	recommendations plus
	*RAS/BRAF* Wild-Type mCRC	*RAS/BRAF* mutant mCRC	recommendation for *dM*MRl*MSI-H* and
			*BRAF* mutant mCRC
Participants	6 experts	6 experts	17 experts

## Recommendations

### Definition of Fit and Unfit Intensive Treatment Patients

Defining fit and unfit patients can help to identify appropriate candidates for intensive therapy. Fitness refers to physical condition, health, and wellbeing, and unfitness refers to cumulative impairment of physiological systems. Fitness status affects patients’ treatment tolerance and survival. Because fitness may improve or deteriorate with treatment, reassessing before every line of treatment is essential.

NCCN colon cancer guidelines version 1.202110 classify mCRC patients as “appropriate for intensive therapy” and “not appropriate for intensive therapy.” A patient appropriate for intensive therapy is defined as “one with good tolerance for this therapy and for whom a high tumor response rate would be potentially beneficial.” ESMO and Pan-Asian ESMO guidelines advise to assess patients as “fit” or “unfit” according to their medical condition. The expert advisor discussed whether to identify patients appropriate for intensive treatment based on clinical diagnosis or radiological diagnosis, debating which of the following factors were more likely to influence fit classification: performance status (ECOG), comorbidity, age, tumor size, sites of metastases, and patient attitude. The general opinion was that none of the three guidelines clearly defines fit and unfit patients, and to avoid confusion, defining fit or unfit was not further addressed in the consensus meetings. The experts also suggested that treatment decisions must consider multiple clinical factors, including age, performance status, organ function, comorbidities, and patient attitude, and cannot be defined by any single factor.

**Table:** 

**Recommendation 1**
Patients should be assessed as fit or unfit based on clinical evaluation. Multiple factors such as age, performance status, organ function, comorbidities, and patient attitude must be considered.

### Consensus on Treatment Goal

Defining treatment goals for mCRC patients is an individualized process dependent on physician and patient values, judgments, and experience. It is also a dynamic process requiring mutual understanding between patients and physicians with regard to patients’ disease status and treatment expectations.

Variables that define treatment goals for mCRC patients are as follows:

- Patient-related factors, such as age and comorbidities
- Tumor-related factors, such as site of metastasis, grade, and hormone-receptor status
- Ongoing objective measures of disease activity, such as survival and response rates
- Impacts of treatment on subjective improvements in quality of life and symptom palliation

Treatment goals in the ESMO and Pan-Asian ESMO guidelines are cure, cytoreduction, and disease control. Treatment goals in the NCCN guidelines are resectable or unresectable (potentially convertible or unconvertible). Consensus on treatment goals for local Taiwanese mCRC patients was reached through discussion and voting.

The experts discussed treatment as a highly complex, dynamic process, with treatment goals only indicated for initial treatment. In current practice with advanced modalities, after the initial treatment, some patients with disease control may become candidates for resection. The majority of experts concorded with the ESMO treatment goals as cure, cytoreduction, and disease control but mentioned that “cure” should be revised to “curative intent” to more accurately express the treatment goal. Experts agreed that dynamic treatment follows after initial treatment. For instance, cytoreduction patients may undergo surgery, continue cytoreduction, or become disease control patients. Hence, further discussion was needed to address the appropriate treatment flow and was the scope for subsequent consensus statements.

Treatment goals for fit patients with initially unresectable metastases are further categorized into two groups: cytoreduction and disease control. ESMO guidelines recognize two types of patients fit for cytoreduction: (1) those for whom intensive treatment is appropriate with the goal of cytoreduction (tumor shrinkage) and conversion to resectable disease; or 2) those who need intensive treatment but will never receive resection or LAT because rapid reduction in tumor burden is needed due to impending clinical threat, organ dysfunction, or severe symptoms. Patients fit for disease control are those with no impending clinical threat for whom intensive treatment is not necessary. Treatment stratification is presented in **Table 4**.

**Table 4:** Definition of cytoreduction and disease control according to ESMO guideline.

Patient’s Classification		Clinical Presentation	Treatment Goal
Fit	Group 1	a) Conversion and achievement of NED	a) Cytoreduction, followed by R0 resection; NED achieved by LAT
		b) Impending clinical threat, impending organ dysfunction and severe (disease-related) symptoms	b) Improvement of symptoms and avoidance of rapid evolution and prolonged survival
	Group 2	• Asymptomatic patients • No impending clinical threat	Disease control and prolonged survival
Unfit		• Best supportive care	Palliative

**Table:** 

**Recommendation 2**
For fit patients with resectable metastatic disease, the treatment goal is curative intent; for fit patients with unresectable metastatic disease, the treatment goal is either cytoreduction or disease control.

### Treatment Consideration of Molecular Profile

Molecular biomarkers play an important role in individualized therapy for mCRC patients. Optimal utilization of molecular biomarker testing is required for patients’ best treatment outcomes.

#### RAS

*RAS* protein is the main regulator of growth factor-induced cell proliferation and survival in both cancer and normal cells. *RAS* is an important gene superfamily, including *KRAS*, *NRAS*, and *HRAS*. *KRAS* is an oncogene coding for the EGFR signaling pathway*. KRAS* is associated with increased cell proliferation, migration, angiogenesis, and survival of CRC tissue. The WT *KRAS* allele is present in 60% of mCRC patients. Determining *KRAS* wild-type status helps to identify tumors with favorable responses to EGFR inhibitors (11) and predicts long-term prognosis (12).

Bokemeyer et al. (13) studied *KRAS* exon 2 wild-type patients from the OPUS study for 26 mutations (referred to as *new RAS*) and additional *KRAS* and *NRAS* codons. New *RAS* mutations were present among 26% of the patients. Patients from the *RAS* wild-type group showed significant improvement by adding cetuximab to FOLFOX4 therapy. A trend toward worse outcomes was also noted among patients with *RAS* mutation when adding cetuximab. Tejpar and Köhne (14) presented another set of results from the OPUS study for patients tested for *KRAS* exons 3 and 4 and *NRAS* exons 2, 3, and 4. Fewer favorable outcomes resulted, and no benefit from adding cetuximab was found among *RAS* mutant population. Further studies showed a significant benefit in all end points among *RAS* wild-type patients by adding cetuximab to the doublet regimen (15, 16).

“Extended *RAS* wild type” was established as a distinct subgroup with significantly better response to anti-EGFR monoclonal antibody. NCCN, ESMO, and Pan-Asian ESMO guidelines strongly recommend extended *RAS* biomarker analysis before treatment with anti-EGFR therapy. *RAS* analysis should include at least *KRAS* exons 2, 3, and 4 (codons 12, 13, 59, 61, 117, and 146) and *NRAS* exons 2, 3, and 4 (codons 12, 13, 59, 61, and 117). Sanger sequencing is a standard method for *RAS* testing but requires at least 10%–25% of *RAS* mutant neoplastic cells in the sample for reliable detection (17). All experts agreed that *RAS* testing is essential before considering the initial treatment.

#### BRAF

The v-Raf murine sarcoma viral oncogene homolog B (*BRAF*) oncogene encodes a serine/tyrosine-kinase downstream to *RAS* in the mitogen-activated protein kinase (MAPK) signaling transduction pathway, which plays an important role in regulating cellular proliferation and survival. *BRAF* mutations are detected almost exclusively in *KRAS*-wild- type CRC and are present in 8.1% of patients with mCRC (18). It is associated with MSI, multiple sites of metastases, more colon tumors (mainly right-sided), higher grade tumors, mucinous histology, adverse histologic features, older age, ECOG performance status ≥2, female gender, and poor survival (19). *BRAF*-mutant CRC has emerged in recent years as a distinct biological entity that is refractory to standard therapy and has a poor prognosis. The mOS for such patients without therapy is around 11 months compared with 35 months for patients with *BRAF* wild-type mCRC (20, 21). Since *BRAF* and EGFR are on the same signaling pathway, *BRAF* mutation is considered to be a negative predictive marker for EGRF antibody treatment response (22, 23). Patients with *BRAF* V600E–mutated metastatic melanoma respond well to *BRAF* inhibitors; however, *BRAF* V600E–mutated mCRC patients respond relatively poorly (only 5%) (21). Current clinical evidence supported expert recommendation to consider *BRAF* testing before initial treatment.

#### MSI

Germline mutations in MMR genes lead to deficient DNA MMR, resulting in DNA MSI phenotype, which also may result from epigenetic silencing of the MLH1 gene. During DNA synthesis, MMR proteins repair base-pair mismatch errors in tandemly repeated sequences called microsatellites. Deficient MMR results in the production of truncated, nonfunctional protein or protein loss results in the MSI phenotype, which is present in 15% of CRC cases but only 4% in the metastatic setting (24). MMR status provides important prognostic and predictive information in patients with early-stage CRC (particularly stage II). MMR-D is associated with both good prognosis (i.e., significantly lower risk of recurrence) and lack of benefit from fluorouracil-based adjuvant therapy (25, 26). Unlike in early-stage disease, no clear evidence is available regarding the prognostic value of MSI-H/dMMR status in mCRC. Mounting evidence suggests that MSI-H/dMMR tumors are less responsive to conventional chemotherapy, but studies have been inconclusive to date, and chemotherapy remains the standard of care for patients with MSI-H/dMMR colorectal cancer (27, 28).

The prominent predictive value of MSI status in CRC has recently emerged following the unprecedented results of immunotherapy with checkpoint inhibitors in MSI-H/dMMR mCRC, including pembrolizumab (29, 30), nivolumab (31, 32), and ipilimumab (32), which have shown long-term survival benefits in these patients. Thus, MSI status has become a crucial biomarker to define patients’ therapeutic options in the metastatic setting.

Current evidence supports MMR/MSI testing before 1L treatment, either by immunohistochemistry (IHC) of MMR protein or PCR-based assay. A panel of microsatellite markers has been validated and recommended as a reference panel for PCR analyses (33). Since patients can be classified as hypermethylated, PCR is more accurate than IHC, yet IHC is more widely available among Taiwan hospitals. Key opinion leaders unanimously recommend both PCR and IHC for MMR/MSI testing.

**Table:** 

**Recommendation 3**
*RAS, *BRAF*, and MMR/MSI testing should be considered before 1L treatment.
For MMR/MSI testing, both PCR and IHC can be adopted.

### Treatment Consideration and Optimal Treatment Choices Among Different Patient Archetypes

#### Optimize Treatment for *RAS*/*BRAF* WT mCRC When Cytoreduction Is the Goal

Cytoreduction (i.e., tumor shrinkage), defined as reduction in tumor volume, correlates highly with prolonged patient survival. When cytoreduction is the goal, the objective response rate (ORR) is considered the primary goal. Better tumor shrinkage allows patients to increase resectability chances, especially to relieve disease-related symptoms in patients with impending threat or severe disease-related symptoms. A meta-analysis by Holch et al. (34) evaluated FIRE-3, CALGB/SWOG 80405, and PEAK. The odds ratio of ORR favored anti-EGFR-based chemotherapy regardless of the primary tumor location (OR: 1.49, 95% CI=1.16–1.0, p=0.002 for left-sided tumor; OR: 1.2, 95% CI= 0.77–1.87, p=0.432 for right-sided tumor) compared to anti-VEGF-based chemotherapy. In addition, anti-EGFR-based chemotherapy showed a higher early tumor shrinkage (ETS) rate (68.2% *vs*. 49.1% in FIRE-3; 64% *vs*. 45% in PEAK) and deeper DpR (48.9% *vs*. 32.3%, p<0.0001 in FIRE-3; 65% *vs*. 46%, p=0.0007 in PEAK) compared with anti-VEGF-based chemotherapy (35).

After weighing the evidence, experts recommended CT doublet plus anti-EGFR as 1L treatment for patients with *RAS*/*BRAF* WT mCRC, regardless of the primary tumor location, when cytoreduction is the goal. As a supportive rationale, experts agreed that data indicated a numerically greater effect from anti-EGFR-based therapy in right-sided tumors. Tumor shrinkage is also much more relevant in liver metastasis to support decisions about the amount of remaining liver tissue to be left. At this stage, tumor shrinkage is more important than tumor location. The experts also agreed that anti-EGFR should be recommended because anti-VEGF is usually withdrawn before surgery.

Due to limitations of Taiwan reimbursement criteria, treatment choices may be different when considering patients’ financial burden. Therefore, for this consensus, financial consideration was excluded from this discussion.

**Table:** 

**Recommendation 4**
Tumor shrinkage is the main consideration when selecting treatment options for *RAS*/*BRAF* WT cytoreduction patients.
CT doublet plus anti-EGFR should be recommended for 1L treatment, regardless of the primary tumor location, when cytoreduction is the goal.

#### Optimal Treatment for *RAS*/*BRAF* Wild-Type mCRC When Disease Control Is the Goal

When disease control is the goal, prolonging survival is considered the primary goal. Four meta-analyses, including the CALGB 80405, FIRE-3, PEAK, and TAILOR studies, have shown significantly better OS, PFS, and ORR with first-line chemotherapy plus anti-EGFR antibodies than with chemotherapy plus anti-VEGF in patients with *RAS* wt left-sided mCRC (36). In contrast, OS was significantly attenuated when using cetuximab/panitumumab in the *RAS* WT right-sided subgroup, and such patients seemed to benefit from chemotherapy plus anti-VEGF.

Currently, NCCN and Pan Asian ESMO guidelines have included tumor location as an important consideration within the treatment algorithm. NCCN guidelines recommend using anti-EGFR agents for treating *RAS* wild-type and left-sided tumors, whereas ESMO guidelines do not consider tumor location. During the meetings, some experts shared that they prefer to consider sidedness for mCRC patients with liver metastases. CT triplet is preferred by some experts for right-sided tumors, and CT doublet is preferred for left-sided tumors.

In summary, when disease control is the treatment goal, most experts suggest that the primary tumor location should be considered, and anti-EGFR shows better treatment outcomes in left-sided *RAS*/*BRAF* WT mCRC because1L treatment is based on current clinical evidence.

Similarly, CT doublet plus anti-VEGF was established as the preferred 1L treatment for right-sided *RAS*/*BRAF* WT mCRC.

**Table:** 

**Recommendation 5**
Primary tumor location should be considered for treatment choices when disease control is the goal.
When disease control is the goal, CT doublet plus anti-EGFR is recommended as the 1L treatment for *RAS*/BRAF WT left-sided tumor, and CT doublet plus anti-VEGF is recommended for *RAS*/*BRAF* WT right-sided tumor.

### Optimal Treatment Option for *RAS* Mutation and *BRAF* Wild-Type Patients

*RAS* mutations are negative predictors of response to anti-EGFR therapy (37). Several large clinical trials, including CO.17, CRYSTAL, OPUS, and PRIME, have elucidated that *KRAS* mutations predict lack of response and clinical benefit from anti-EGFR mAbs in patients with mCRC (16, 38–44). The phase III FIRE-3 trial randomized 592 patients with *KRAS* exon 2 WT in a head-to-head comparison of first-line cetuximab *versus* bevacizumab in combination with FOLFIRI (16). Of 407 *KRAS* exon 2 WT tumors that could be sequenced, 65 (16%) harbored other *RAS* mutations. Analysis of 65 patients with other *RAS* mutations revealed a lower response rate (38% *vs*. 58%), PFS (6.1 *vs*. 12.2 months), and OS (16.4 *vs*. 20.6months) in the cetuximab arm compared to bevacizumab arm. The phase II PEAK study randomized 285 patients with *KRAS* exon 2 WT tumors between FOLFOX plus panitumumab and FOLFOX6 plus bevacizumab in the first-line setting (40). Expanded *RAS* testing showed that 51 patients (23.1%) harbored a non-*KRAS* exon 2 *RAS* mutation. Patients with *RAS* mutant tumors showed negative effects of panitumumab treatment (PFS, 7.8 *vs*. 8.9months; HR, 1.39) compared to bevacizumab treatment.

The BECOM study was a phase II, randomized controlled trial evaluating the efficacy of bevacizumab plus mFOLFOX6 vs. mFOLFOX6 alone as first-line therapy of *RAS* mutant unresectable colorectal liver metastases (44). In 121 patients, the bevacizumab plus mFOLFOX6 group demonstrated better R0 resection rates for liver metastases (22.3% *vs*. 5.8%, P<0.01), objective response rates (54.5% *vs*. 36.7%, P<0.01), median PFS (9.5 *vs*. 5.6 months, P<0.01), and median OS (25.7 *vs*. 20.5 months, P=0.03) than the mFOLFOX6 alone group.

ESMO, NCCN, and Pan-Asian ESMO treatment guidelines recommend CT doublet plus anti-VEGF antibody for *RAS* mutation patients. Targeted therapy is the preferred treatment and is currently reimbursed by Taiwan National Health Insurance. The experts recommended CT doublet plus anti-VEGF as the 1L treatment for *RAS* mutant patients, but more intensive CT triplet plus anti-VEGF should be considered for cytoreduction purposes.

**Table:** 

**Recommendation 6**
CT doublet plus anti-VEGF is recommended as 1L treatment for *RAS* mutant patients, but CT triplet plus anti-VEGF should also be considered for cytoreduction purposes.

### Optimal Treatment Option for *BRAF* Mutation Patients

#### Optimize Initial Treatment for Patients With *RAS* WT/*BRAF* Mutant mCRC

Prognosis is poor for patients with mCRC harboring *BRAF* mutations (42). Good response rates (90%), median PFS (12.8 months), and OS (30.9 months) were reported in a subgroup of 10 patients with *BRAF*-mutant tumors treated with FOLFOXIRI plus bevacizumab (*post hoc* analysis) (39). These results were confirmed in a prospective study in 214 patients, including 15 with *BRAF*-mutant tumors (45). A 60% response and median PFS and OS of 9.2 and 24.1 months, respectively, were found in a single-arm phase II trial of mCRC patients treated with first-line folinic acid, fluorouracil, oxaliplatin, and irinotecan (FOLFOXIRI)-bevacizumab. Pooling of retrospective and prospective results showed median PFS and OS of 11.8 and 24.1 months, respectively. Similarly, subgroup analysis showed that 16 patients with *BRAF*-mutant tumors treated with FOLFOXIRI plus bevacizumab had an ORR of 56% and median PFS and OS of 7.5 and 19.0 months, respectively, compared with 12 patients treated with FOLFIRI plus bevacizumab who had slightly lower ORR of 42% (OR: 1.82, 95% CI=0.38–8.78) and shorter median PFS of 5.5 months (HR: 0.57, 95% CI=0.27–1.23) and median OS of 10.7 months (HR: 0.54, 95% CI=0.24–1.20) (41). Based on these results, ESMO and Pan-Asian ESMO guidelines recommended triplet chemotherapy plus bevacizumab as the standard of care for first-line treatment of BRAF-mutant CRC. The ANCHOR CRC single-arm, phase II study (43) in first-line *BRAF*V600E mCRC patients, recently published at World Congress on GI Cancer 2020, aimed to investigate the efficacy of triplet therapy with *BRAF* inhibitor encorafenib, MEK inhibitor binimetinib, combined with cetuximab in treatment-naïve patients with *RAS* WT/*BRAF* V600E mutant mCRC, in which 51% of the patients had peritoneal metastases. The ORR was 50% (95% CI= 33.8–66.2), and 85% of patients had decreased tumor size. Median PFS was 4.9 months (95%CI, 4.4–8.1). Adverse events were consistent with those observed in prior studies with this triplet combination (46).

Based on currently available evidence, the experts reckoned that data from the TRIBE study were unconvincing as it was subgroup analysis from a small sample. However, HRs at each efficacy endpoint favored the FOLFOXIRI plus bevacizumab arm. Considering the high toxicity of FOLFOXIRI, most Taiwanese patients do not tolerate it, and CT doublet plus bevacizumab is considered another treatment option. Experts agreed that CT triplet/doublet plus anti-VEGF should be recommended for 1L *BRAF* mutant patients. In the ANCHOR study, encorafenib plusbinimetinib and cetuximab showed promising efficacy and tolerable toxicity in first-line treatment of *BRAF* mutant mCRC patients, emphasizing that this patient population had high median age (67 years) and disease burden (56% ECOG 1, 78% ≥ 2 metastases site, 51% peritoneal metastasis). Although a phase II, single-arm study has not yet been completed, experts agreed not to include it as a recommendation for first-line treatment for *BRAF* mutant, and it will be revisited after more data become available.

**Table:** 

**Recommendation 7**
CT doublet/triplet plus anti-VEGF is recommended as first-line treatment for *BRAF* mutant patients.

#### Optimize Treatment for Patients With Refractory *RAS* WT/*BRAF* Mutant mCRC

The open-label, phase 3 trial BEACON CRC study (21, 46) included 665 patients with *BRAF* V600E–mutated mCRC who experienced disease progression after one or two previous regimens. Patients were randomized to receive encorafenib, binimetinib, and cetuximab (triplet-therapy group); encorafenib and cetuximab (doublet-therapy group); or the investigators’ choice of cetuximab and irinotecan or cetuximab and FOLFIRI (folinic acid, fluorouracil, and irinotecan) (control group) in a 1:1:1 ratio. The mOS was 9.3 months in both the triplet- and doublet-therapy groups *vs*. 5.4 months in the control group (P<0.001 vs. control). The ORRs were 27% and 20% in the triplet- and doublet-therapy groups, respectively, and 2% in the control group (P<0.001 *vs*. control). The mOS in the doublet-therapy group was 8.4 months (P<0.001 *vs*. control). Updated results revealed that the doublet-therapy regimen showed similar overall efficacy and well-tolerated safety profile as the triplet-therapy regimen. Encorafenib in combination with cetuximab was approved by the FDA (https://www.accessdata.fda.gov/drugsatfda_docs/label/2020/210496s006lbl.pdf) and EMA (information_en.pdf) in *BRAF*V600E-mutant mCRC after prior therapy.

During consensus meetings, encorafenib plus cetuximab ± binimetinib was recommended by the experts as both triplet and doublet regimens with demonstrated superiority compared to controls, and clinicians could select regimens based on each patient’s condition. Different expert opinions toward the doublet or triplet regimen included preference for the doublet regimen because the FDA and NCCN have included this regimen in recent updates, and only limited benefits were seen by adding MEKi to the regimen. Triplet regimens were preferred by other experts because of personal clinical experience with the *BRAF* and MEK inhibitor in combination with anti-EGFR. *BRAF* mutant patients were believed to progress rapidly, and triplet therapy may be beneficial in disease control. Because encorafenib and binimetinib were not available in Taiwan, the recommendation was adjusted to BRAFi plus anti-EGFR ± MEKi. Overall, consensus inclined toward using BRAFi plus anti-EGFR ± MEKi as second-line treatment for mCRC patients carrying *BRAF* mutation.

A brief summary of trials evaluating the role of targeted therapy in patients with *BRAF* mutated mCRC is shown in **Table 5**.

**Table 5:** Trials evaluating role of targeted therapy in *BRAF* mutated patients.

Study	N	Phase	Line	Arms	ORR	Median PFS	Median OS
Yaeger et al. (47)	15	Pilot	2nd or 3rd	Vemurafenib plus Panitumumab	13%	3.2 months	7.6 months
Hyman et al. (45)	27	II	2nd above	Vemurafenib plus Cetuximab	4%	3.7months	7.1 months
Kopetz et al. (21)	21	II	2nd above	Vemurafenib	5%	2.1 months	7.7 months
Stintzing et al. (48)	48	III	1st	FOLFIRI plus cetuximab *vs* FOLFIRI plus bevacizumab	52.2% *vs* 40% (p=0.56)	6.6 months in both arms (HR=0.84, p=0.56)	12.3 *vs* 13.7 months (HR=0.79, p = 0.45)
Loupakis et al. (49)	15	II	1st	FOLFOXIRI plus bevacizumab	60%	9.2 months	24.1 months
Lopez-Crapez and Thezenas (50)	5	II	1	FOLFIRINOX plus Cetuximab	80%	6.1 months	21.3 months
Geissler and Knorrenschield (51); Geissler and Marc Martens (52)	16	II	1st	mFOLFOXIRI plus panitumumab *vs* FOLFOXIRI	85.7% *vs* 22.2% (p=0.041)	6.5 months *vs* 6.1 months	8.0 months *vs* 9.0 months
Grothey and Taeib (43)	41	II	1st	Encorafenib plus Binimetinib plus Cetuximab	50%	4.9 months	–
Kopetz et al. (50, 51)	665	III	2nd or 3rd	Encorafenib plus Binimetinib plus Cetuximab *vs* Encorafenib plus Cetuximab *vs* Investigators’ choice of either cetuximab and irinotecan or cetuximab and FOLFIRI	27%: 20%: 2% (P<0.001 for both triplet *vs* control and doublet *vs* control group)	4.5 months: 4.3 months: 1.5 months (HR: 0.42, 95% CI=0.33–0.53 for triplet *vs* control group; HR: 0.44, 95% CI=0.35–0.55 for doublet *vs* control group)	9.3 months; 9.3 months; 5.9 months (HR:0.52, 95% CI=0.47–0.75 for triplet *vs* control group; HR: 0.61, 95% CI=0.48–0.77 for doublet *vs* control group)
Corcoran et al. (53)	142	I dose-escalation	Any line	Dabrafenib (D) plus panitumumab (P) ± MEK inhibition with trametinib (T)	ORR in D+T+P, T+P, D+P, and D + T were 21%, 0%, 10%, and 7%, respectively	PFS in D+T+P, T+P, D+P, and D + T were 4.2, 2.6, 3.5, and 3.5 months, respectively	OS in D+T+P, T+P, and D+P arms were 9.1, 8.2, and 13.2 months, respectively

**Table:** 

**Recommendation 8**
BRAFi plus anti-EGFR ± MEKi is recommended as 2L treatment for *BRAF* mutant patients.

### Optimal Treatment Option for Patients With MSI-H/dMMR mCRC

Antitumor activity of the checkpoint inhibitor was observed in MSI-H CRC but not in MSS CRC. Based on data from phase II KEYNOTE-016 of pembrolizumab in patients with MSI- H CRC and MSS CRC, the ORR of the CRC MMR-deficient (MSI-H) arms was 57% (95% CI, 39%–73%), whereas the ORR in the MSS CRC arm was 0% (95% CI, 0%–13%).

In the phase II KEYNOTE-16429 (n=124) and Checkmate-142 monotherapy cohort (31) (n=74), pembrolizumab and nivolumab monotherapy demonstrated around 30% ORR in patients with treatment-refractory MSI-H/dMMR mCRC. Results with combination ipilimumab plus nivolumab (n=119) were even more remarkable, with a response rate of 55% (95% CI, 45.2–63.8) and 12-month PFS and survival rates of 71% and 85%, respectively (32).

In Checkmate-142, nivolumab plus low-dose ipilimumab was investigated in first-line MSI- H/dMMR mCRC (54). Two-year follow-up results showed that this combination provided high and durable clinical benefits with 69% ORR (13% CR) without reaching the duration of response. The 24-month PFS and OS rates were 74% and 79%, respectively.

In 2020, the FDA approved pembrolizumab for first-line treatment of unresectable dMMR/MSI-H mCRC patients based on data from the phase 3 KEYNOTE-177 trial, in which 307 treatment-naïve MSI-H/dMMR mCRC patients were enrolled (55). In that study, treatment with pembrolizumab significantly reduced the risk of disease progression or death by 40% (HR, 0.60; 95% CI, 0.45–0.80; *P* =.0004) *versus* standard-of-care chemotherapy (2). Additionally, the PD-1 inhibitor had more than double PFS *versus* chemotherapy at 16.5 months (95% CI, 5.4–32.4) *versus* 8.2 months (95% CI, 6.1–10.2), respectively. Additional results presented during the 2020 ASCO Virtual Scientific Program (56) showed that the 12-month PFS rate was 55% in the pembrolizumab arm and 37% in the chemotherapy arm; the 24-month PFS rates were 48.3% and 18.6%, respectively. The ORR was 43.8% with pembrolizumab and 33.1% with chemotherapy. Responses were more durable with the PD-1 inhibitor than chemotherapy. The median duration of response had not been reached in the pembrolizumab arm compared with 10.6 months in the chemotherapy arm.

Although pembrolizumab monotherapy has met its primary endpoint of improving PFS compared to chemotherapy in KEYNOTE-177, most experts do not recommend listing the immune checkpoint inhibitor in first-line treatment for MSI-H/dMMR since it lacks OS data and cannot explain having higher PD risk than chemotherapy (29.4% *vs*. 12.3%). Some experts believe that patients who are both MSI-H and *BRAF* mutant seem to have better outcomes with the immune checkpoint inhibitor. Overall, immune checkpoint inhibitor monotherapy is an optional choice for first- and second-line treatment for dMMR/MSI-H patients. Experts emphasized that, similar to consensus for *BRAF* mutant patients, the immune checkpoint inhibitor should be described as better than pembrolizumab.

**Table:** 

**Figure 2:**
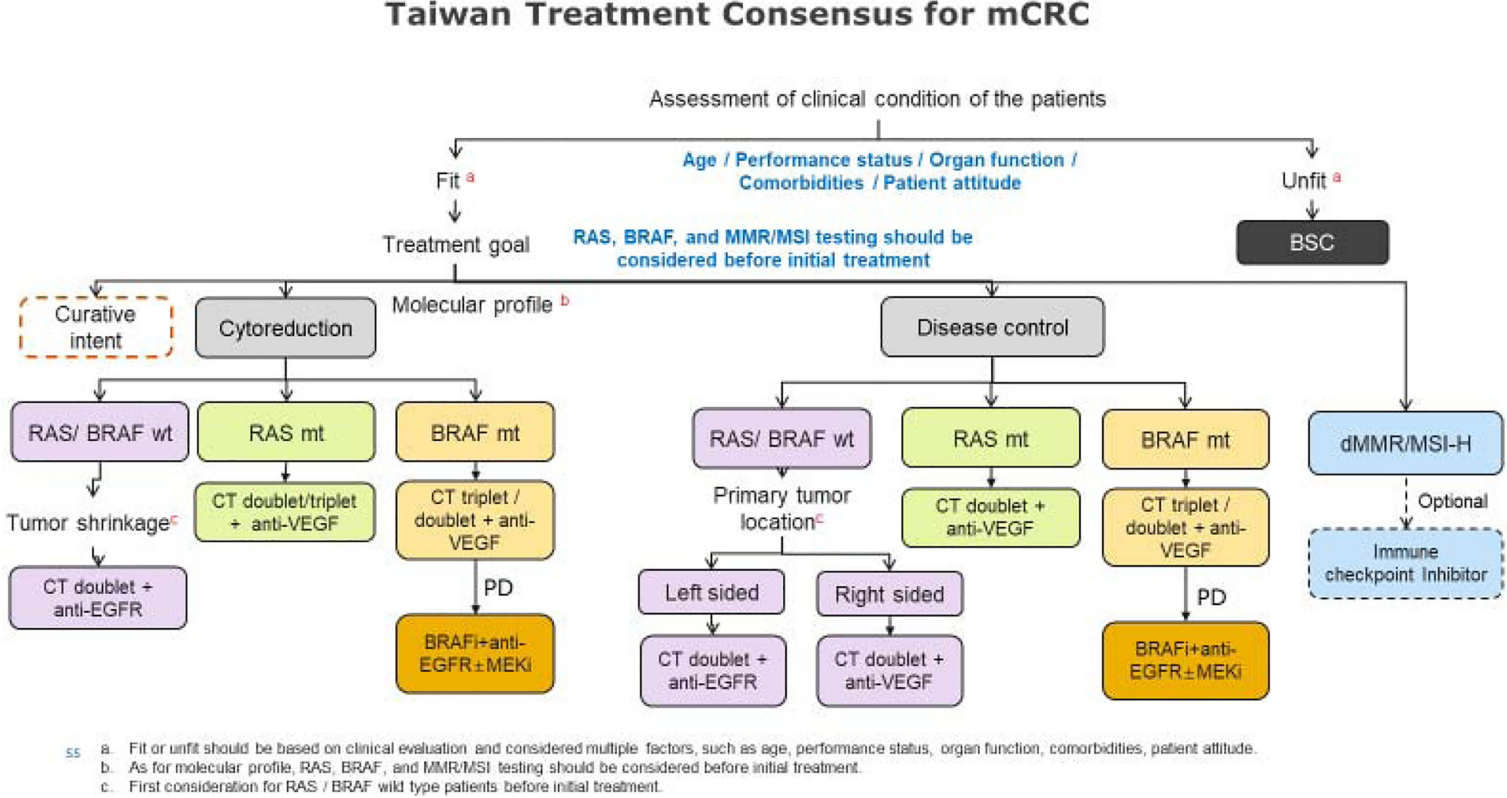
Taiwan treatment consensus of CRC.

**Recommendation 9**
In addition to CT plus target therapy, the immuno-checkpoint inhibitor is an alternative option for any line of treatment for patients with dMMR/MSI-H mCRC.
The Taiwan treatment consensus for mCRC is shown in **Figure 2**.

## Conclusion

CRC is recognized as a heterogeneous disease requiring individualized management strategies for its subtypes. This consensus is second in the Taiwan Society of Colon and Rectal Surgeons (TSCRS) Consensus series to address the unmet gaps in guideline recommendations in lieu of Taiwanese mCRC management. It represents the outcome of key opinion leaders’ thought exchange over 3 years on certain unclear aspects in international guidelines. International guidelines were considered as a framework, and discussion addressed gaps in recommendations for advising local Taiwanese clinical practice. The ultimate goal was to improve Taiwanese patient outcomes. As more data emerge and efficacy of newer agents is established, these recommendations will be refined during further meetings.

## Author Contributions

Conception and design: H-HC, T-WK, C-WH, J-KJ, C-CC, Y-YH, H-WT, B-WL, Y-HL, Y-LS, H-CH, F-CK, Y-HC, JL, B-RL, Y-YC, and J-YW. Acquisition of data: H-HC, T-WK, C-WH, J-KJ, C-CC, Y-YH, H-WT, B-WL, Y-HL, Y-LS, H-CH, F-CK, Y-HC, JL, B-RL, Y-YC, and J-YW. Analysis and interpretation of data: H-HC, T-WK, C-WH, J-KJ, C-CC, Y-YH, H-WT, B-WL, Y-HL, Y-LS, H-CH, F-CK, Y-HC, JL, B-RL, Y-YC, and J-YW. Drafting of the manuscript: J-YW. Final approval of the manuscript: H-HC, T-WK, C-WH, J-KJ, C-CC, Y-YH, H-WT, B-WL, Y-HL, Y-LS, H-CH, F-CK, Y-HC, JL, B-RL, Y-YC, and J-YW. All authors contributed to the article and approved the submitted version.

## Funding

The writing grant was supported by Merck KGaA. The funder was not involved in the study design, collection, analysis, interpretation of data, the writing of this article or the decision to submit it for publication.

## Conflict of Interest

The authors declare that the research was conducted in the absence of any commercial or financial relationships that could be construed as a potential conflict of interest.

## Publisher’s Note

All claims expressed in this article are solely those of the authors and do not necessarily represent those of their affiliated organizations, or those of the publisher, the editors and the reviewers. Any product that may be evaluated in this article, or claim that may be made by its manufacturer, is not guaranteed or endorsed by the publisher.
